# The Salford Union Infirmary

**Published:** 1909-12-18

**Authors:** F. Townson

**Affiliations:** Clerk to the Guardians. Union Offices, Eccles New Road, Salford


### EDITOR'S LETTER-BOX.

### THE SALFORD UNION INFIRMARY.

*To the Editor of* The Hospital.

Sir,—Adverting to the report on poor-law infirmaries by Sir Henry Burdett which appeared in your issue of December 4, I am directed to forward you the following copy of a resolution adopted by the Guardians of this Union at their meeting to-day on the recommendation of their Infirmary Committee, namely :

"Resolved : That this committee, having had their attention called to the article by Sir Henry Burdett in The Hospital of December 4, declare the same to be a scurrilous and unwarranted attack on the members of this Board, present and past, who for upwards of twenty years have devoted their time and ability in the interests of the sick poor of Salford. The committee feel strongly that the comparison of the Salford Union Infirmary with institutions of the Charles Dickens type casts a serious reflection and unmerited aspersion on the institution and those responsible for its upkeep and management, and the committee would desire to take this opportunity of stating that they would willingly invite the fullest possible investigation by any unprejudiced person, or persons, with a view to a proper comparison being made with other poor-law or general hospitals, and that they would render every facility in their power to this end."

I am directed to ask you if you will be good enough to insert the above in your next issue.

Yours faithfully,

F. Townson,

Clerk to the Guardians.

Union Offices, Eccles New Road, Salford,
December 10, 1909.

∴ It is reported that sixteen Guardians were present at the meeting, of whom eight voted for the resolution, five voted against it, and three did not vote at all. We welcome this letter, however, in the hope that much good may result from the appointment of a medical superintendent.—Ed. The Hospital.

